# Patients’ and Psychologists’ Preferences for Feedback Reports on Expected Mental Health Treatment Outcomes: A Discrete-Choice Experiment

**DOI:** 10.1007/s10488-022-01194-2

**Published:** 2022-04-15

**Authors:** Loes Hilhorst, Jip van der Stappen, Joran Lokkerbol, Mickaël Hiligsmann, Anna H. Risseeuw, Bea G. Tiemens

**Affiliations:** 1 Behavioural Science Institute, Radboud University, Nijmegen, The Netherlands; 2 Centre of Economic Evaluation, Trimbos Institute (Netherlands Institute of Mental Health), Utrecht, The Netherlands; 3 Department of Health Services Research, CAPHRI Care & Public Health Research Institute, Maastricht University, Maastricht, The Netherlands; 4 MIND Ypsilon, Amersfoort, The Netherlands; 5 Pro Persona Research, Renkum, The Netherlands

**Keywords:** Routine outcome monitoring, Expected treatment outcome, Patient preference, Psychologist preference, Discrete choice experiment, Choice

## Abstract

In recent years, there has been an increasing focus on routine outcome monitoring (ROM) to provide feedback on patient progress during mental health treatment, with some systems also predicting the expected treatment outcome. The aim of this study was to elicit patients’ and psychologists’ preferences regarding how ROM system-generated feedback reports should display predicted treatment outcomes. In a discrete-choice experiment, participants were asked 12–13 times to choose between two ways of displaying an expected treatment outcome. The choices varied in four different attributes: representation, outcome, predictors, and advice. A conditional logistic regression was used to estimate participants’ preferences. A total of 104 participants (68 patients and 36 psychologists) completed the questionnaire. Participants preferred feedback reports on expected treatment outcome that included: (a) both text and images, (b) a continuous outcome or an outcome that is expressed in terms of a probability, (c) specific predictors, and (d) specific advice. For both patients and psychologists, specific predictors appeared to be most important, specific advice was second most important, a continuous outcome or a probability was third most important, and feedback that includes both text and images was fourth in importance. The ranking in importance of both the attributes and the attribute levels was identical for patients and psychologists. This suggests that, as long as the report is understandable to the patient, psychologists and patients can use the same ROM feedback report, eliminating the need for ROM administrators to develop different versions.

## Introduction

Although effective treatments for mental health problems are available, the symptoms and functioning of a substantial proportion of patients do not improve, or even worsen (de Beurs et al., 2015). In fact, therapists often do not recognize patients’ stagnation or deterioration (Hatfield et al., 2010). This may mean that the treatment does not suit the patient or his or her problems. For this reason, in recent years there has been increasing focus on evaluating patients’ treatment progress using routine outcome monitoring (ROM).

With ROM, progress in a patient's symptoms and functioning is monitored in a structured way using standardized questionnaires. Based on these scores, the ROM system creates a report of patients' current symptoms and functioning, as well as the course of their illness to date (Kendrick et al., 2016; Lutz et al., 2015). These reports will be called *feedback reports* throughout this article. It has been shown that using these feedback reports can have a positive influence on treatment outcome (De Jong et al., 2021; Kendrick et al., 2016; Lambert et al., 2018; Lutz, et al., 2015; Schibbye et al., 2014). Some studies show that feedback reports could further enhance treatment effectiveness if they were accessible to both the therapist and the patient (Fortney et al., 2017; Gondek et al., 2016; Moltu et al., 2016; Solstad et al., 2020).

However, it appears that little use is made of ROM in everyday practice (Jensen-Doss et al., 2018). Even when ROM scores are collected by asking the patient to fill out questionnaires, therapists do not always review and use the feedback reports within therapy, and ROM systems are not always well-implemented (Bickman et al., 2016; Lewis et al., 2019). A possible explanation for this could be that various factors hamper the implementation of ROM systems. For example, therapists often do not have time to utilize such a system in their day-to-day work, or there is too little trust among therapists and patients about what would happen with the results (Boswell et al., 2013). Research has shown that the extent to which a therapist is committed to using feedback reports is related to the amount of progress being made in alleviating a patient's symptoms (De Jong et al., 2012). Research, therefore, should also focus on ways to enhance therapists’ willingness to utilize feedback reports.

Therapists’ willingness to use feedback reports may be related to the content of these reports and the way in which they are displayed. For example, feedback reports that are too extensive can cause confusion and lead to frustration (Hovland & Moltu, 2019). In some cases, the addition of visual features (e.g., line graphs of progress over time) seems to make feedback reports more intuitive, thereby allowing the therapist to understand it more easily (Hovland & Moltu, 2019; Hovland et al., 2020; Moltu et al., 2016). Therefore, the design of feedback reports should align with therapists’ preferences, so that they will be motivated to use these feedback reports in treatment.

Furthermore, some research has shown that patients want to discuss their ROM scores with their therapist and that they prefer a direct way of discussing scores and exploring the underlying reasons for their responses (Solstad et al., 2020). This, in combination with the finding that feedback reports could enhance treatment effectiveness more if they were accessible to both the therapist and the patient (Fortney et al., 2017; Gondek et al., 2016; Moltu et al., 2016; Solstad et al., 2020), shows that it is important that feedback reports are designed to be understandable and easy to work with for patients. This highlights the need to consider both the therapist and the patient when developing feedback reports.

In addition to the design, the content of feedback reports is influential. Most ROM systems reflect only patients' current symptoms and functioning and the course of their illness to date (Lutz et al., 2015). However, there are systems that predict treatment outcomes for a specific patient. For instance, based on a patient’s ROM scores and characteristics, some systems show expected recovery curves (Lambert et al., 2018; Lutz et al., 2019). So far, such systems have hardly ever been used in the Netherlands (Tiemens & Van Sonsbeek, 2017). Predicting treatment outcomes could help treatment providers further adapt treatment so that it better matches particular patients’ characteristics, situation, and problems (Lutz et al., 2019). To make feedback reports that include predicted treatment outcomes attractive to both therapists and patients, their preferences need to be taken into account.

We used a discrete-choice experiment (DCE) to quantitatively investigate psychologists’ and patients’ preferences for feedback reports that include expected treatment outcomes. DCEs are commonly used in healthcare to elicit preferences (De Bekker-Grob et al., 2012; Soekhai et al., 2018), and they are increasingly being used in mental health care (e.g. Becker et al., 2016; Lokkerbol Geomini et al., 2018; Lokkerbol Van Voorthuijsen et al., 2018; Tünneßen et al., 2020). In this case, a DCE is a technique that solicits latent preferences regarding various attributes of feedback reports about expected treatment outcomes. Using various combinations of attribute levels, it is possible to elicit preferences for certain attributes relative to other attributes (De Bekker-Grob et al., 2012). Participants are repeatedly given two choices and asked to make judgments about their preference for one of the choices (De Bekker-Grob et al., 2012).

Although some DCEs have been used in mental health care, psychologists’ and patients’ preferences for feedback reports on expected treatment outcome have not been well investigated. Research has been limited to preferences about feedback reports on patients’ current symptoms and functioning (Hovland & Moltu, 2019; Hovland et al., 2020; Moltu et al., 2016; Solstad et al., 2019, 2020). For example, Moltu et al. (2016) found that patients preferred feedback reports that included information about external factors that could potentially affect treatment outcomes (e.g. sleeping pattern) and therapists indicated a preference for information about underlying aspects of patients’ symptoms, such as their level of energy. In addition, Solstad et al., (2020) found that patients preferred feedback reports that included information about therapeutic alliance. To our knowledge, none of these studies have assessed feedback reports in terms of how outcome predictions are presented. Therefore, the research question of the current study was: *What are the preferences of both psychologists and patients for feedback reports on expected treatment outcome in mental health care and what are the similarities and differences between the preferences of psychologists and patients?* The aim of this study was to use a DCE to elicit these preferences.

## Methods

### Participants

Using *the rule of thumb* (De Bekker-Grob et al., 2015), we calculated a minimum sample size in advance. *The rule of thumb* is defined as 500 * [the maximum number of attribute levels (3)]/[the number of choice tasks (12) * the number of alternatives (2)], which indicated that the study should include a minimum of 63 participants in order to estimate main effects. To be able to do subgroup analyses, aiming to double that sample size is recommended (De Bekker-Grob et al., 2015), resulting in a targeted sample size between 63 and 126.

Patients were included if they (a) were currently receiving therapy for a mental health problem or had received therapy in the previous 12 months, and (b) were 18 years old or older. For one third of the participating patients, the question about received therapy contained an error. Consequently, for one third of the participating patients, we did not obtain confirmation that they had actually received therapy. However, all of the remaining patients confirmed that they were currently receiving therapy or had done so in the previous 12 months. Moreover, all participants were recruited in the same way, via recruitment advertisements that explicitly stated the inclusion criteria, so we expect that all the participants had received therapy, despite the lack of confirmation. Therefore, no patients were excluded.

Psychologists were included if they were currently working as a clinical psychologist or had done so in the previous 12 months. Every participating psychologist confirmed being or having been employed as a psychologist, thus none of them were excluded. In the introduction of the questionnaire, psychologists were asked to only participate if they had worked with feedback reports, but there was no question to confirm this. In addition, participating patients were not asked if they had ever received feedback reports on their symptomatology in their treatment. Therefore, it is uncertain whether each participant had experience with feedback reports.

### Discrete-Choice Experiment

Using a discrete-choice experiment (DCE), we investigated psychologists’ and patients’ preferences regarding feedback reports on expected treatment outcome. The DCE explained to participants that they would be provided with treatment outcome predictions for themselves (if they were patients) or for a fictitious patient (if they were psychologists) that would be constructed in several different ways, and that they would be asked repeatedly to indicate the way they preferred. They were given several choice tasks, each of which contained two alternatives for feedback reports providing a prediction of treatment outcome. They were asked to choose their preferred alternative (Prediction A or Prediction B) for each choice set. Each alternative presented the same hypothetical prediction and contained the same attributes, but they differed according to attribute levels. The attributes were characteristics of the prediction (e.g., how the prediction should be represented or whether the prediction included advice about how to continue the treatment), and the attribute levels were variations of these characteristics (e.g., for the attribute *representation*, the attribute levels were *text* versus *text and images*, and for the attribute *advice*, the attribute levels were *no advice*, *general advice* or *specific advice*).

To identify attributes and attribute levels, we used: conversations with a psychologist, a DCE expert, and patient representatives; multiple consultations among the researchers; and a review of the literature. This process produced four attributes, each of which had either two or three levels. Table 1 shows the final list of attributes, their attribute levels, and a description of each attribute level. Table 2 shows the images used in the choice tasks for the level *text and images* of the attribute *representation*.

**Table 1:** Attributes and Attribute Levels

Attribute and levels	Variable	Content
*Representation*		
Text	TEXT	–
Text and images	Reference level	–
*Outcome*		
Dichotomous	DICHOTOMOUS	This prediction means that the symptoms are not expected to improve within this treatment
Continuous	CONTINUOUS	This prediction means that the patient belongs to the group in which the symptoms of 20 to 40 out of 100 clients will improve within this treatment
Probability	Reference level	This prediction means that at the end of treatment the patient will have a score between 70 and 80 on a questionnaire about his or her symptoms. This is a high score and means that the patient will still experience a lot of discomfort due to the complaints
*Predictors*		
No	NO PRED	No information is available about the factors that influenced this prediction
General	GEN. PRED	The factors that influenced this prediction are the following:
		– The sleeping pattern
		– The patient’s level of social support
		– The course of symptoms in the first half of treatment
Specific	Reference level	The factors that influenced this prediction are the following:
		– The sleeping pattern is disturbed. This can influence the treatment outcome negatively
		– The patient has a lot of social support. This can influence the treatment outcome positively
		– The symptoms have not improved in the first half of the treatment. This can influence the treatment outcome negatively
*Advice*		
No	NO ADVICE	No advice is available
General	GEN. ADVICE	It is advised that the patient and the therapist continue to discuss whether the treatment fits the needs of the patient and the experienced problems
Specific	Reference level	It is advised that the patient and the psychologist continue to discuss whether the treatment fits well, whether the patient feels understood and whether a positive bond is experienced by both. See if the goals of the patient and the psychologist match and if the treatment method is appropriate. Investigate whether factors such as sleep patterns and patient-experienced social support influence treatment success and incorporate them into the formulation of the treatment plan

**Table 2:**
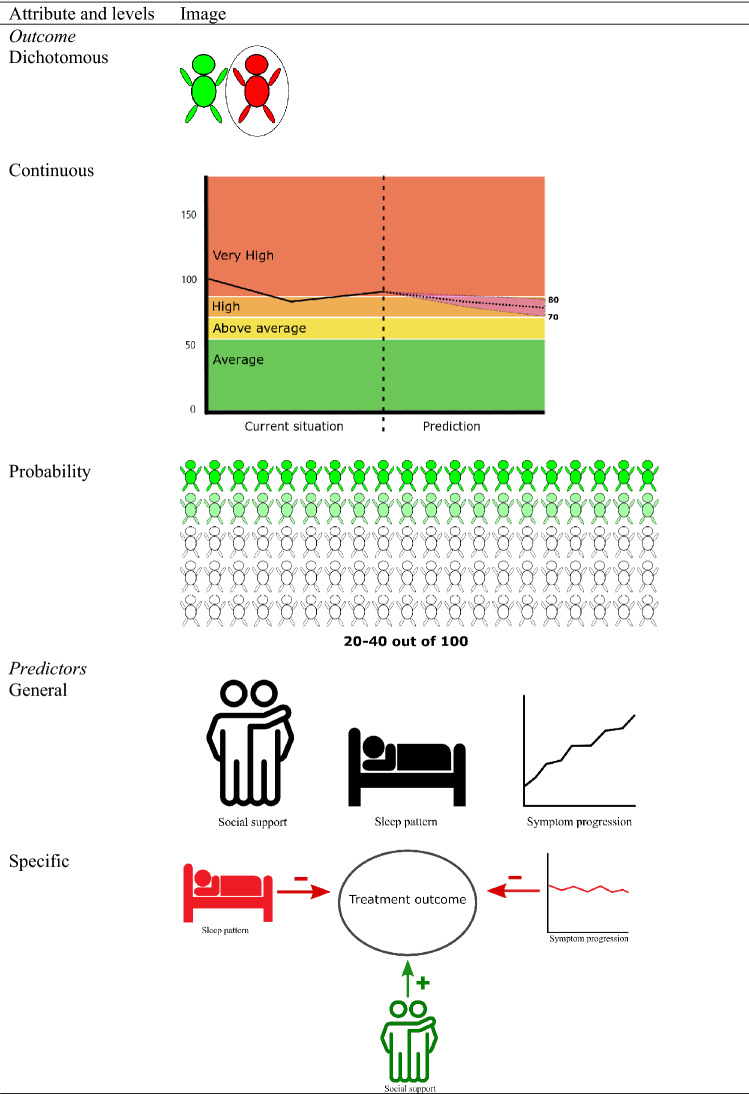
Images that were used in case of the attribute *representation*, level *text and images*

### Attributes and Attribute Levels

One way in which feedback reports can differ is in how the prediction is represented. Feedback reports can be displayed graphically, as an audio file, in writing, or as a combination of these forms (Harmon et al., 2005; Lambert, 2007). We investigated whether the participants preferred one or the other of these methods of feedback, although feedback in the form of an audio file was excluded due to practical reasons.

Another feature of feedback reports is the unit of measurement in which the predicted outcome is described. According to Gigerenzer, (2011), a description that includes natural frequencies (e.g., in the case of a specific patient; 74 out of 100 patients improve) would be easier to understand than would proportions. Additionally, a continuous description, in the form of a score on a symptom questionnaire, might be preferable because it is often used in feedback reports (Kendrick et al., 2016; Lambert, 2007). Within feedback reports, dichotomous outcome measures are also used, for example those based on Jacobson and Truax's, (1991) Reliable Change Index for indicating a reliable and clinically significant change. We investigated whether the participants preferred a dichotomous outcome, a continuous outcome, or one that is expressed in terms of a probability.

Another feature on which feedback reports may differ is whether they include information about the main predictors of the expected treatment outcome. These predictors can be potential risks or protective factors, which therapists may want to bring up within a treatment. Moltu et al., (2016) have shown that therapists like to be informed about potential risk factors associated with treatment outcome. This suggests that they might like to be informed about the main predictors of the expected treatment outcome. Additionally, previous research has shown that patients are often uncertain about the goals of ROM when they fill out questionnaires, and they would like to be more informed about how the ROM will be used (Solstad et al., 2019). This finding suggests that patients may prefer transparency about the methods of ROM, and thus about how an expected treatment outcome was predicted and what factors influenced it. Our study examined whether participants preferred information about the predictors of an expected treatment outcome.

The final feature this study considered was whether the prediction included advice about how to proceed in treatment. Research has shown that a predicted treatment outcome is relevant to a therapist only when that prediction is followed by recommendations for clinical strategies to adapt treatment to the patients’ needs (Lutz et al., 2019). For example, Simon et al., (2012) assessed the effectiveness of clinical support tools in which they combined information about progress in treatment with problem-solving tools. They found that these methods improved treatment outcomes. Moltu et al., (2016) showed that both patients and therapists value patient ownership of the treatment process. Giving advice can provide more insight into the treatment process and thus potentially contribute to this ownership. On the other hand, advice can potentially make the patient feel that his or her opinions about the therapy are undervalued. Thus, it is also possible that feedback including advice could actually detract from the patient’s sense of ownership. Furthermore, Simon et al., (2012) found that therapists did not always find clinical support tools useful. We investigated whether the participants preferred feedback reports that included advice about how to proceed in treatment.

### Choice Tasks

**Fig. 1:**
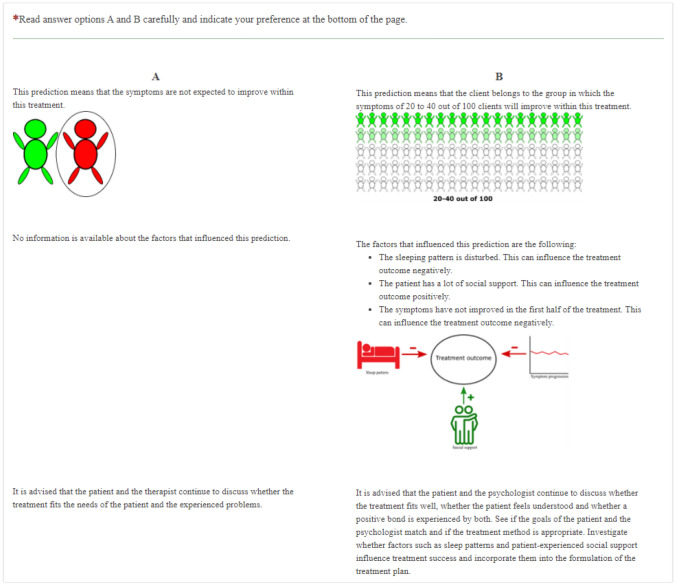
An example of one the choice tasks

To ensure that participants were not required to assess all possible combinations (2,916) of the attributes and attribute levels, Ngene software (http://choice-metrics.com/) provided a subset of the attributes and their levels. An orthogonal design containing 36 choice tasks was designed, each of which contained two alternatives for feedback reports on expected treatment outcome. One of the 36 choice tasks was removed because it contained two identical options. However, because a questionnaire consisting of 35 choice tasks was still too excessive, we used *blocking* to shorten it (Reed Johnson et al., 2013). Blocks contain a portion of the choice tasks. Respondents are assigned to a random block and therefore do not have to complete the entire set of questions. The use of block design is recommended and allows for better statistical efficiency than having only one version for all respondents (Reed Johnson et al., 2013). Three blocks were developed, each containing 11 or 12 choice tasks, with a test–retest reliability question (a duplicate of the third choice-task) added to each block. Each participant was randomly allocated to one of the three blocks and therefore completed 12 or 13 choice tasks, consistent with common practice when using a DCE (Soekhai et al., 2018). Each alternative presented the same hypothetical prediction (the client's symptoms would not improve within treatment), but each used different modalities. Figure 1 shows an example of one of the choice tasks.

### Measure Administration

Participants were recruited via advertisements on the social media channels (Facebook and LinkedIn) of the Netherlands Institute of Mental Health and Addiction (Trimbos Institute) and through the researchers' private networks (by e-mail). In a short text on social media or in an email, readers were asked to participate if they were working as a psychologist and had experience with ROM in the past 12 months, or if they had received psychological treatment in the past 12 months. They were further told that they would receive a five-euro voucher for participating, that participation would be anonymous, and that the questionnaire would take approximately 15 to 20 min to complete. This was followed by a link to a letter containing information about the questionnaire, the purpose of the research, data processing, and privacy. After the participants had agreed to the details included in their information letter and had given informed consent, they were randomly allocated to receive one of the three blocks.

### Measures

**Fig. 2:**
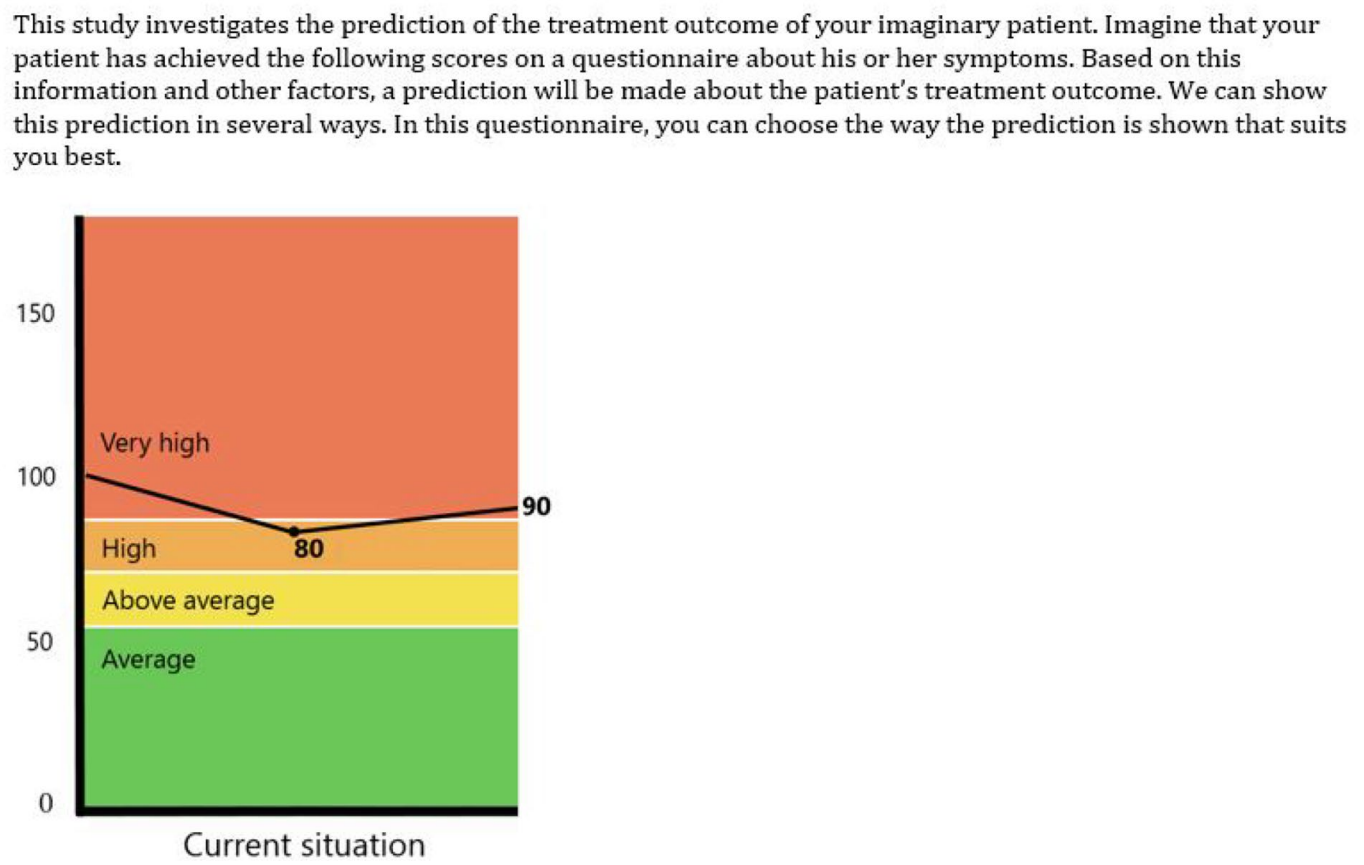
Situation sketch that was shown to the psychologists

Each block started with a sketch of a situation (Fig. 2). The participants were asked to imagine that they (if they were patients) or a fictious patient (if they were psychologists) had achieved the scores that were shown on a symptom questionnaire. They were further told that, based on this and other information, a prediction about treatment outcome could be made, and that in each choice task they should choose the manner of displaying this prediction that they preferred. This was followed by an example question and the 12 or 13 choice tasks. Then participants were asked about their age, gender, and level of education. Finally, the psychologists were asked to indicate the number of months that they had been working as psychologists, and the patients were asked to indicate the number of months they had been treated for a mental health problem. This last question was used to further check the eligibility of the respondents. In one of the three questionnaires for the patients, this question contained an error. Consequently, for one third of the participating patients, we did not obtain confirmation that they had actually received therapy.

The survey was developed for online delivery using LimeSurvey, and the respondents completed the questionnaires via LimeSurvey (https://www.limesurvey.org/). Prior to the study, the authors evaluated the questionnaire for accuracy, functionality, and routing. In addition, a client representative and a psychologist evaluated the questionnaire for its comprehensibility.

The Ethics Committee of the Faculty of Social Sciences (ECSW) at Radboud University approved the study. Data collection took place between May and June 2020.

### Statistical Analysis

The results were analyzed using a conditional logistic regression, which was carried out in Nlogit (http://www.limdep.com/products/nlogit/). It was assumed that the preference *V* for an alternative *j* was represented as an addition as shown in the following formula:

$$\begin{gathered} {\text{V}}_{{\text{j}}} = \, \beta_{{1}} *{\text{ TEXT }} + \, \beta_{{2}} *{\text{ DICHOTOMOUS }} + \, \beta_{{3}} *{\text{CONTINUOUS }} + \, \beta_{{4}} *{\text{NO}}.{\text{PRED}}. \, + \hfill \\ \, \beta_{{5}} *{\text{GEN}}.{\text{PRED}}. \, + \, \beta_{{6}} *{\text{NO}}.{\text{ADVICE }} + \, \beta_{{7}} *{\text{GEN}}.{\text{ADVICE }} + \, \varepsilon_{{\text{j}}} \hfill \\ \end{gathered}$$

The attribute levels *text and images*, *probability*, *specific predictors*, and *specific advice* were the reference levels, therefore, they are omitted here. *β*_*1*_ to *β*_*7*_ were the preference coefficients for each attribute level. Using effect coding, we modeled the variables so that the mean influence of an attribute on the preference for an alternative was zero. The preference coefficients were thus compared to zero, which was the mean effect of the attribute. A positive preference coefficient means that the corresponding attribute level had a positive effect on the preference for a certain alternative. In case a coefficient had a negative preference, this attribute level had a negative influence on this preference. The preference coefficients were significant when zero was not in the confidence interval. The attribute levels are equal to 0 or 1, depending on whether this attribute level is applied in the alternative. Finally, *ε*_*j*_ stands for standard error.

Additionally, the conditional relative importance (CRI) was calculated for each attribute. This is a percentage that indicates how important the attribute was for choosing a certain alternative. For each attribute, the CRI was calculated by dividing the difference between the highest preference coefficient and the lowest preference coefficient by the sum of the differences between the highest preference coefficient and the lowest preference coefficient of each attribute. The formula for calculating attribute *x* was as follows:

$$\left( {{\text{highest }}\beta {\text{ attribute x }}{-}{\text{ lowest }}\beta {\text{ attribute x}}} \right) \, / \, \left( {\sum \, \left( {{\text{highest }}\beta {\text{ attribute }}{-}{\text{ lowest }}\beta {\text{ attribute}}} \right)_{{{1} \ldots {4}}} } \right) \, *{ 1}00\%$$

Finally, an additional analysis was conducted to compare the preferences of the patients and psychologists. To this end, we estimated a joint model using interaction terms. Interaction terms that were significantly different from zero indicated that the patients’ preferences were different from those of the psychologists.

## Results

### Participants

Of the 475 participants who began the questionnaire, 104 of them (22%) completed it. Incomplete results were excluded from the analysis. Block 1 was completed by 20 patients and 14 psychologists, Block 2 was completed by 28 patients and 13 psychologists, and Block 3 was completed by 20 patients and 9 psychologists. Table 3 shows the respondents’ characteristics. Each of the psychologists (*n* = 36) who completed the questionnaire had been working as a psychologist during the previous 12 months. As a result of the error in the question about treatment history on one of the questionnaires, we were unable to confirm that one-third of the patients had actually received therapy. All of the remaining patients confirmed that they were currently receiving therapy or had done so in the previous 12 months. Of the 104 participants in the final sample, 82 (79%) of them answered the test–retest question (the duplicate of the third question) identically to the third question, consistent with previous DCE studies (Lokkerbol Geomini et al., 2018; Lokkerbol Van Voorthuijsen et al., 2018).

**Table 3:** Respondents’ Characteristics

Characteristic	Total		Patients (*n* = 68)		Psychologists (*n* = 36)	
	*n*	Percentage	*n*	Percentage	*n*	Percentage
*Gender*						
Male	13	13%	7	10%	6	17%
Female	89	86%	59	87%	30	83%
Undisclosed	2	2%	2	3%	0	0%
Age in years*						
18–24	24	23%	23	34%	1	3%
25–30	31	30%	20	30%	11	31%
31–40	26	25%	10	15%	16	44%
41–50	9	9%	5	7%	4	11%
51–60	11	11%	8	12%	3	8%
61–70	2	2%	1	2%	1	3%
*Level of education*						
Primary education	1	1%	1	2%	0	0%
Secondary education						
Low	2	2%	2	3%	0	0%
Middle	7	7%	7	10%	0	0%
High	8	8%	8	12%	0	0%
Higher education						
Low	11	11%	11	16%	0	0%
Middle	17	16%	17	25%	0	0%
High	58	56%	22	32%	36	100%

*One participant’s age is unknown because of an error in this person’s response to the question about age

### Participants’ Preferences

**Fig. 3:**
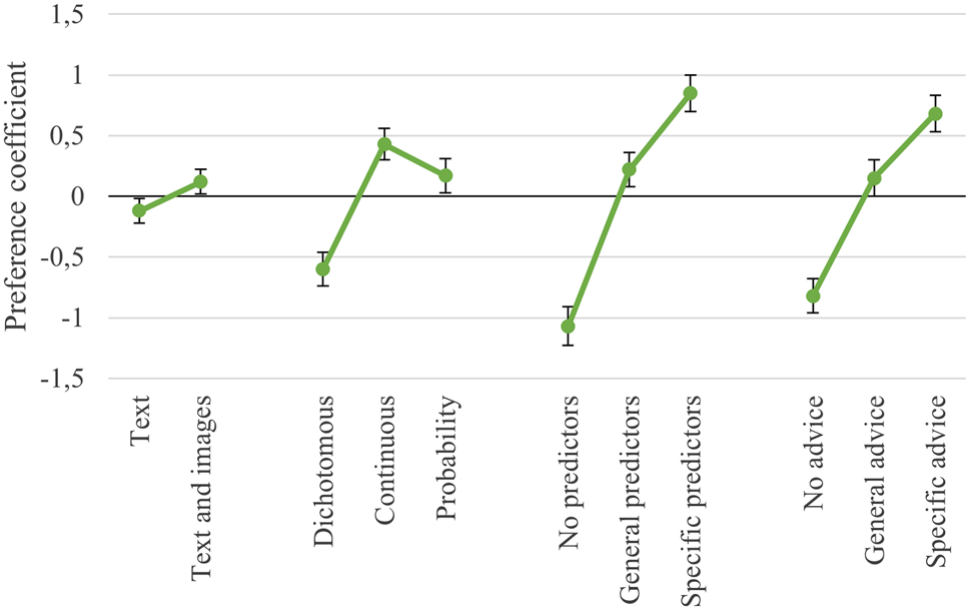
Preference Coefficients for each Attribute Level

The results from the conditional logistic regression analysis are shown in Table 4 and Fig. 3. The fact that the preference coefficients are positive and the confidence intervals do not overlap indicates that the participants preferred feedback reports with text and images over text only. Furthermore, patients significantly preferred a continuous outcome and one that was expressed as a probability over a dichotomous outcome. There was no significant difference in preference for feedback reports with a continuous outcome versus an outcome expressed as a probability. Finally, for both attributes, participants significantly preferred specific information about the predictors and specific advice over general information or no information.

**Table 4:** Results from the Conditional Logistic Regression Analysis

Attributes and levels	Preference coefficient β (95% CI)	*SD*	*p*-value	CRI
*Representation*				5.0%
Text	− .12 (− .22, − .02)	.05	.023	
Reference level: Text and images	.12 (.02, .22)	.05	.023	
*Outcome*				22.0%
Dichotomous	− .60 (− .74, − .46)	.07	< .001	
Continuous	.43 (.29, .56)	.07	< .001	
Reference level: Probability	.17 (.03, .31)	.07	.015	
*Predictors*				40.9%
No	− 1.07 (− 1.24, − .91)	.08	< .001	
General	.22 (.09, .36)	.07	.001	
Reference level: Specific	.85 (.70, 1.00)	.08	< .001	
*Advice*				32.0%
No	− .82 (− .97, − .68)	.07	< .001	
General	.15 (.00, .30)	.08	.058	
Reference level: Specific	.68 (.53, .83)	.08	< .001	

Number of observations: 1214; log-likelihood: − 611.97

The CRI for the attribute *predictors* (40.9%) was highest of all of the attributes, which indicated that when respondents chose between two alternative feedback reports, the attribute *predictors* was the most important of them all. The results indicate that participants were willing to accept feedback reports that did not fully match their preferences if the feedback reports included specific information about the predictors. The smallest CRI was 5.0%. This indicates that the attribute *representation* was the least important to the respondents when they chose between two feedback reports on an expected treatment outcome.

### Subgroup Analysis: Patients Versus Psychologists

**Fig. 4:**
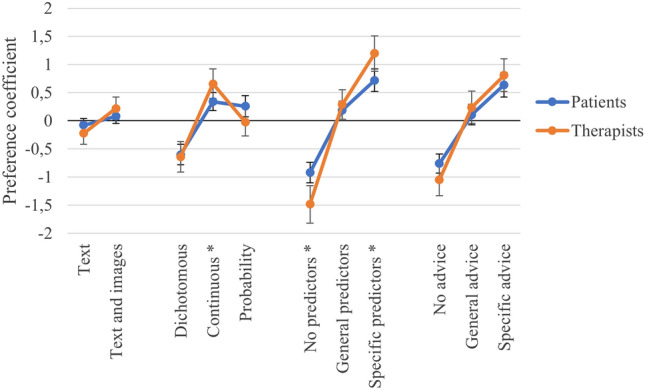
Psychologists’ and Patients’ Preference Coefficients for each Attribute Level. *Significant difference in preference between psychologists and patients

Table 5 and Fig. 4 show the results from the subgroup analysis comparing patients with psychologists. Overall, the two groups showed similar results. There were, however, significant differences between the two groups in their preferences for three of the attribute levels: continuous outcome, no predictors, and specific predictors. Regarding outcomes depicted as a probability, the psychologists had a significantly greater preference for continuous outcomes than the patients. In addition, compared to the patients, the psychologists had a significantly greater dislike for feedback reports without predictors, and a significantly stronger preference for feedback reports that included specific predictors.

**Table 5:** Differences between Psychologists’ and Patients’ Preferences for Different Feedback Reports on Expected Treatment Outcome

Attributes and levels	Patients	Psychologists	*p*-value
Number of participants	68	36	
*Representation*	CRI 4.1%	CRI 7.0%	
Text	− .08 (− .21, .04)	− .22 (− .42, − .02)	.211
Reference level: Text and images	.08 (− .04, .21)	.22 (.02, .42)	.211
*Outcome*	CRI 22.5%	CRI 20.7%	
Dichotomous	− .60 (− .77, − .42)	− .64 (− .91, − .37)	.725
Continuous	.34 (.19, .50)	.66 (.40, .92)	.038
Reference level: Probability	.26 (.07, .45)	− .02 (− .27, .24)	.077
*Predictors*	CRI 39.7%%	CRI 42.7%	
No	− .92 (− 1.11, − .74)	− 1.48 (− 1.81, − 1.14)	.002
General	.19 (.03, .35)	.29 (.03, .54)	.479
Reference level: Specific	.72 (.53, .92)	1.20 (.88, 1.51)	.012
*Advice*	CRI 33.7%	CRI 29.6%	
No	− .76 (− .92, − .59)	− 1.05 (− 1.33, − .77)	.076
General	.11 (− .07, .29)	.24 (− .05, .53)	.455
Reference level: Specific	.64 (.42, .86)	.81 (.52, 1.10)	.345

Number of observations: 1214; log-likelihood: − 602.01

## Discussion

This study investigated psychologists’ and patients’ preferences regarding various designs of feedback reports on expected treatment outcomes. For the participants, the choice between no predictors, general predictors, or specific predictors was the most important. The participants preferred specific information about the predictors over general information or no information at all. The second most important was the choice between specific advice, general advice, or no advice. The results suggest that with regard to advice being offered, the participants favored specific information over general information or no information at all. The third most important was the choice between continuous outcomes versus dichotomous outcomes versus outcomes being expressed as a probability. They preferred that feedback reports contain a continuous outcome or one that was stated in terms of a probability instead of a dichotomous outcome. Finally, the participants preferred feedback reports that included images over reports that consisted only of text, but this choice was the least important. This finding is noteworthy because it is often argued that images make feedback reports easier to understand, and this is the reason why feedback reports usually contain images (Harmon et al., 2005; Hovland et al., 2020; Lambert, 2007).

A subgroup analysis indicated that psychologists preferred feedback reports with a continuous outcome more than patients did. Compared to the patients, the psychologists also had a stronger dislike for feedback reports that did not include predictors, and had a stronger preference for feedback reports that included specific predictors. Nevertheless, the ranking in importance of both the attributes and the attribute levels was identical for the patients and the psychologists, despite differences in educational attainment between the two subgroups. Such agreement between psychologists and patients could be beneficial for clinical practice. In the case of shared decision-making, patients are involved in the choices made regarding their treatment, and this is associated positively with treatment outcome (Schauer et al., 2007). The finding that psychologists and patients preferred the same designs of feedback reports suggests that feedback reports on expected treatment outcomes could be helpful for shared decision-making regarding progress in therapy. This also suggests that it might not be necessary to consider the differences between patients’ and psychologists’ preferences when developing feedback reports on predicted treatment outcomes, allowing a single format to be implemented.

This format should include specific information about the main predictors of the expected treatment outcome, and specific advice. Furthermore, the predicted treatment outcome should be continuous or expressed in terms of a probability. Participants preferred a prediction that included images over a text-only prediction, indicating that feedback reports on expected treatment outcome should include images. However, this was the least important to the participants, which implies that adding graphics to predicted treatment outcomes should be given the least priority. Because most ROM systems cannot currently predict a treatment outcome, evaluation of how to develop the preferred style of feedback report should proceed within the context of clinical practice. This evaluation should also include the technical feasibility of predicting treatment outcomes.

This study used a specific choice of images, but there are multiple ways to visualize data in this context (Grossman & Masterson Creber, 2018). Therefore, future research should include other ways of displaying a similar prediction, to see if these might have a different effect, or possibly affect the relative importance of the choice to include images in a report.

Research has shown that patients are often unaware of the rationale for ROM; therefore, they do not always complete the questionnaires that they have been asked to fill out (Solstad et al., 2019). Developing more comprehensive predictions about treatment outcome, requires more information from patients. Because patients seem to place more value on the addition of in-depth information about predictors and advice than they do on predicted treatment outcomes, this might be useful to motivate patients to complete questionnaires. Therefore, providers should clarify during treatment how ROM can help patients with their treatment.

This study did not evaluate whether the content of the prediction influences the psychologists’ and patients’ preferences. In the current study, a prediction was used in which the symptoms of a fictitious patient did not improve during the course of the treatment. The content was, therefore, negative. Psychologists’ and patients’ preferences for how positive versus negative predictions are represented might differ. Research has shown that negative feedback is more effective in changing therapists’ behavior than positive feedback is (Sapyta et al., 2005). Peterson & Fagan, (2020) even found that the content of a feedback report (patient deteriorates, approaches remission, or is not progressing) influenced which features of the feedback report caused behavior change within treatment among therapists. Furthermore, research has shown that therapists are more likely to recommend psychological counseling to a fictitious patient when that patient's emotional state is formulated negatively than when the exact same state is formulated positively (e.g., neuroticism versus emotional stability) (Brandstätter & Mücke, 2009). In addition, receiving feedback about negative results during ROM can be frustrating for patients, because negative feelings can be magnified when the patient sees such results (Börjesson & Boström, 2019). Patients might therefore prefer less specific information related to negative feedback to minimize negative feelings. For these reasons, follow-up research should aim to determine whether the content of the prediction influences preferences for feedback reports on expected treatment outcomes.

Finally, it is possible that a prediction about a treatment outcome affects a patient's or a psychologist's optimism about the treatment. Patients' positive expectations about the outcome of the treatment is often seen as one of the primary mechanisms underlying positive therapeutic change (Greenberg et al., 2006). Although there has been little research on this topic, several qualitative studies have shown that a psychologist’s optimism seems to have a positive influence on the patient's treatment outcome (Bartholomew et al., 2019; Coppock et al., 2010). Future research should, therefore, focus on the effects that feedback reports on a predicted treatment outcome have on both the psychologist's and the patient's sense of optimism. One might expect that a negative prediction would lead to decreased optimism, but that a positive prediction would lead to an increase in optimism. In this way, a prediction itself might indirectly influence the treatment outcome through the mediating factor of optimism.

### Strengths and Limitations

To the best of our knowledge, this is the first study to investigate psychologists’ and patients’ preferences for ways of designing feedback reports on expected treatment outcomes. Within an exploratory design, the study elicited the preferences of both patients and psychologists. The results lay the foundation for follow-up research and clinical applications.

There are some limitations to this study that should be noted. First, social media was used to recruit participants, which resulted in a non-representative sample of mostly young, female respondents. As a consequence, the results cannot be generalized to male and older patients and psychologists. One aspect which the skewed age distribution might have influenced, for example, is psychologists' preference for feedback including advice. Older psychologists may have been practicing longer and therefore have more work experience. In addition, they are less trained in the use of feedback from questionnaires. This could reduce their desire for advice, so the preference in this study could have been different if the average age of the participating psychologists had been higher. Indeed, research by Skovholt and Ronnestad, (1992) shows that novice psychologists rely primarily on external expertise, and that more experienced psychologists tend to rely on their own competence. To be able to generalize the results to male and female participants of all ages, follow-up research should include a participant group with representative age and gender distribution. In addition, only 22% of all the participants completed the questionnaire. Also, we are unable to compare completers with non-completers, which limits the generalizability of the results. Another limitation of the online recruitment of participants was our lack of information about the participants’ opinion on the content and quality of the feedback alternatives and how well they understood the questionnaire. The test–retest stability, however, was high, which suggests that participants’ understanding was sufficient. Besides, prior to the study, a client representative and a psychologist evaluated the questionnaire for its comprehensibility. Future research should aim to use the questionnaire in clinical settings, which might promote respondents’ involvement, confirm that their understanding of the questionnaire is adequate and investigate their opinions on the content and quality of the feedback alternatives.

Second, we were unable to determine in which area of mental health treatment the participating psychologists worked. Thus, we do not know whether the sample was representative of the different types of psychologists, or whether there was an under- or overrepresentation of psychologists in a specific area or with a certain therapeutic orientation. It is possible that psychologists working in different areas have differing preferences for feedback reports on expected treatment outcomes. For example, research has shown that psychologists who deliver short-term treatments are more likely to expect that mental health problems will change as a result of factors external to treatment than psychologists who see patients for an extended period of time (Bolter et al., 1990). Psychologists who deliver short-term treatments might have a greater need for information about external factors that are potentially contributing to the outcome than psychologists who deliver long-term treatments. Such potential differences in preferences among psychologists working in different areas should be investigated in follow-up research.

No information was available about the characteristics of the patients who participated, such as their diagnosis, treatment history, or the amount of social support they were receiving. It is not known, therefore, whether this sample was representative of all people with a mental health problem. Different patient characteristics are associated with different symptoms, which require different treatments. These differences might result in patients having different preferences for feedback reports about their expected treatment outcome. De Jong et al., (2017), for example, found differences in the effects of feedback reports on the treatment outcome of patients with a Cluster B personality disorder versus those with a Cluster C disorder. The presence and type of personality disorder may also affect preferences for different ways of designing feedback reports on expected treatment outcome. Follow-up research should, therefore, investigate such potential differences in preferences among patients with different characteristics.

Furthermore, during the recruitment of participants, psychologists were asked only to participate if they had worked with feedback reports before, but the questionnaire did not contain a question to confirm this. Participating patients were also not asked if they had ever received feedback reports on their symptomatology in their treatment. Consequently, there is no certainty whether each participant had experience with feedback reports and how this may have influenced their preferences for feedback reports on expected treatment outcomes. Therefore, future research should include whether participants have experience with feedback reports and whether this influences their preferences for feedback reports on expected treatment outcomes.

Finally, it should also be noted that the situation sketch that we showed to the participants prior to presenting them with the choice tasks might have influenced their preferences. The feedback depicting the continuous outcome contained the same type of image as the one shown in the situation sketch. It is possible, therefore, that the participants preferred this type of representation because they had previously seen this type of image, and it was familiar to them. There might also have been a priming effect. Through priming, a person might unconsciously react in a different way to a stimulus that he or she has seen previously (Tulving & Schacter, 1990). Follow-up research could eliminate the effect that the situation sketch might have had by choosing a situation sketch that does not resemble one of the images used in the choice tasks.

## Conclusion

This study suggests that both patients and psychologists prefer feedback reports on expected treatment outcomes that have the following characteristics: They should include (a) both text and images, (b) a continuous outcome or an outcome that is expressed in terms of a probability, (c) specific predictors, and (d) specific advice. For both patients and psychologists, specific predictors appeared to be most important; specific advice was second most important; a continuous outcome or a probability was third most important, and feedback that includes both text and images was fourth in importance. The ranking in importance of both the attributes and the attribute levels was identical for patients and psychologists. This suggests that when developing predicted treatment outcomes, it should not be necessary to consider differences between patients and psychologists in their preferences; only one format would need to be developed. These results should be taken into account when developing tools for predicting treatment outcomes.
